# *N*-(2-{[5-Bromo-2-(morpholin-4-yl)pyrimidin-4-yl]sulfan­yl}-4-meth­oxy­phen­yl)-4-chloro­benzene­sulfonamide

**DOI:** 10.1107/S1600536812036689

**Published:** 2012-08-31

**Authors:** Mohan Kumar, L. Mallesha, M. A. Sridhar, Kamini Kapoor, Vivek K. Gupta, Rajni Kant

**Affiliations:** a Department of Studies in Physics, Manasagangotri, University of Mysore, Mysore 570 006, India; b PG Department of Studies in Chemistry, JSS College of Arts, Commerce and Science, Ooty Road, Mysore 570 025, India; c X-ray Crystallography Laboratory, Post-Graduate Department of Physics & Electronics, University of Jammu, Jammu Tawi 180 006, India

## Abstract

In the title compound, C_21_H_20_BrClN_4_O_4_S_2_, the benzene rings bridged by the sulfonamide group are tilted relative to each other by a dihedral angle of 70.2 (1)° and the dihedral angle between the sulfur-bridged pyrimidine and benzene rings is 69.5 (1)°. The mol­ecular conformation is stabilized by a weak intra­molecular π–π stacking inter­action between the pyrimidine and the 4-chloro­benzene rings [centroid–centroid distance = 3.978 (2) Å]. The morpholine ring adopts a chair conformation. In the crystal, mol­ecules are linked into inversion dimers by pairs of C—H⋯N hydrogen bonds and these dimers are further connected by N—H⋯O hydrogen bonds, forming a tape along the *a* axis.

## Related literature

For related structures of sulfonamides, see: Rodrigues *et al.* (2011 ▶); Akkurt *et al.* (2011 ▶); Kant *et al.* (2012 ▶). For bond-length data, see: Allen *et al.* (1987 ▶). For ring conformations, see: Duax & Norton (1975 ▶).
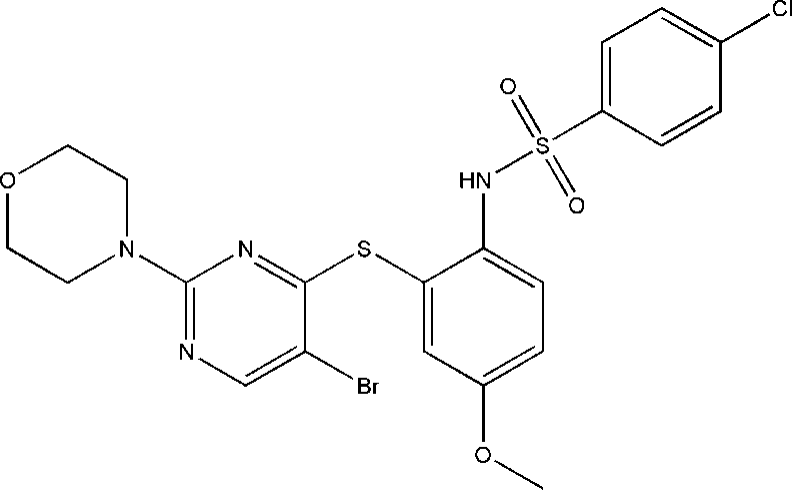


## Experimental

### 

#### Crystal data

C_21_H_20_BrClN_4_O_4_S_2_
*M* *_r_* = 571.89
Monoclinic, 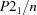

*a* = 10.0311 (3) Å
*b* = 17.3096 (6) Å
*c* = 13.9223 (4) Å
β = 91.829 (3)°
*V* = 2416.16 (13) Å^3^
*Z* = 4
Mo *K*α radiation
μ = 2.02 mm^−1^
*T* = 293 K
0.3 × 0.2 × 0.2 mm

#### Data collection

Oxford Diffraction Xcalibur Sapphire3 diffractometer
Absorption correction: multi-scan (*CrysAlis PRO*; Oxford Diffraction, 2010 ▶) *T*_min_ = 0.438, *T*_max_ = 0.668
26979 measured reflections
4744 independent reflections
3408 reflections with *I* > 2σ(*I*)
*R*_int_ = 0.048

#### Refinement

*R*[*F* ^2^ > 2σ(*F* ^2^)] = 0.045
*wR*(*F* ^2^) = 0.117
*S* = 1.03
4744 reflections
299 parameters
H-atom parameters constrained
Δρ_max_ = 0.42 e Å^−3^
Δρ_min_ = −0.65 e Å^−3^

### 

Data collection: *CrysAlis PRO* (Oxford Diffraction, 2010 ▶); cell refinement: *CrysAlis PRO*; data reduction: *CrysAlis RED* (Oxford Diffraction, 2010 ▶); program(s) used to solve structure: *SHELXS97* (Sheldrick, 2008 ▶); program(s) used to refine structure: *SHELXL97* (Sheldrick, 2008 ▶); molecular graphics: *ORTEP-3* (Farrugia, 1997 ▶); software used to prepare material for publication: *PLATON* (Spek, 2009 ▶).

## Supplementary Material

Crystal structure: contains datablock(s) I, global. DOI: 10.1107/S1600536812036689/is5183sup1.cif

Structure factors: contains datablock(s) I. DOI: 10.1107/S1600536812036689/is5183Isup2.hkl

Supplementary material file. DOI: 10.1107/S1600536812036689/is5183Isup3.cml

Additional supplementary materials:  crystallographic information; 3D view; checkCIF report

## Figures and Tables

**Table 1:** Hydrogen-bond geometry (Å, °)

*D*—H⋯*A*	*D*—H	H⋯*A*	*D*⋯*A*	*D*—H⋯*A*
N7—H7⋯O25^i^	0.86	2.09	2.849 (4)	148
C20—H20⋯N19^ii^	0.93	2.52	3.352 (5)	149

Symmetry codes: (i) 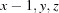; (ii) 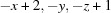.

**Fig. 1.**
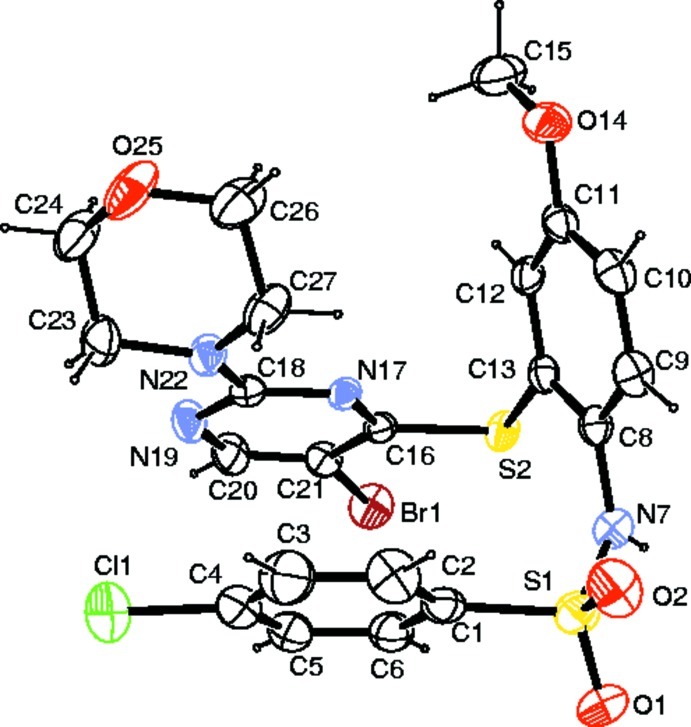
ORTEP view of the title molecule with the atom-labeling scheme. The displacement ellipsoids are drawn at the 40% probability level. H atoms are shown as small spheres of arbitrary radii.

**Fig. 2.**
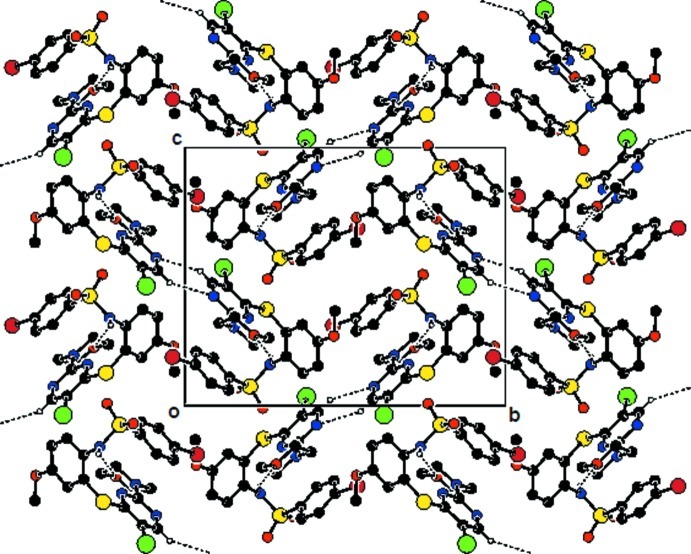
A molecular packing view of the title compound down the a axis, showing intermolecular interactions. For clarity, hydrogen atoms which are not involved in hydrogen bonding have been omitted.

## supplementary crystallographic information

### Comment

Bond lengths and angles in the title compound (Fig. 1) have normal values (Allen *et al.*, 1987) and are comparable with the similar crystal structures (Rodrigues *et al.*, 2011; Akkurt *et al.*, 2011; Kant *et al.*, 2012). The molecule is twisted at atom S1 with a C1—S1—N7—C8 torsion angle of 60.8 (3)°. The morpholine ring is exhibiting a chair conformation [asymmetry parameters are: ΔC2(N22—C23) = 2.53; ΔCs(N22—O25) = 1/5; Duax & Norton, 1975]. The two benzene rings (C1—C6/C8—C13) are tilted relative to each other by 70.2 (1)° and the dihedral angle between the sulfur bridged pyrimidine and benzene rings is 69.5 (1)°. The molecular conformation is stabilized by a weak intramolecular stacking interaction between the pyrimidine and the 4-chloro benzene rings [centroid–centroid distance = 3.978 (2) Å, interplanar spacing = 3.340 Å, and centroid shift = 2.16 Å]. In the crystal, molecules are linked into dimers by pairs of C20—H20···N19 hydrogen bonds and these dimers are further linked by N7—H7···O25 hydrogen bonds (Table 1 and Fig. 2).

### Experimental

The reaction of *N*-[2-(5-bromo-2-chloro-pyrimidin-4-ylsulfanyl)-4-methoxy-phenyl]-4-chloro-benzenesulfonamide (5.22 g, 0.01 mol) with morpholine (0.88 g, 0.01) were carried out in the presence of triethylamine and the reaction mixture was allowed to stir at room temperature for 6–7 h in dry dichloromethane. The progress of the reaction was monitored by TLC. Upon completion, the solvent was removed under reduced pressure and residue was extracted with ethyl acetate. The compound was purified by successive recrystallization from methanol (yield 80%, m.p. 462–464 K)

### Refinement

All H atoms were positioned geometrically and were treated as riding on their parent C and N atoms, with C—H distances of 0.93–0.97 Å and N—H distance of 0.86 Å, and with *U*_iso_(H) = 1.2*U*_eq_(C, N) or 1.5*U*_eq_(methyl C).

### Crystal data

**Table:**

C_21_H_20_BrClN_4_O_4_S_2_	*F*(000) = 1160
*M**_r_* = 571.89	*D*_x_ = 1.572 Mg m^−^^3^
Monoclinic, *P*2_1_/*n*	Mo *K*α radiation, λ = 0.71073 Å
Hall symbol: -P 2yn	Cell parameters from 9194 reflections
*a* = 10.0311 (3) Å	θ = 3.5–29.0°
*b* = 17.3096 (6) Å	µ = 2.02 mm^−^^1^
*c* = 13.9223 (4) Å	*T* = 293 K
β = 91.829 (3)°	Block, white
*V* = 2416.16 (13) Å^3^	0.3 × 0.2 × 0.2 mm
*Z* = 4	

### Data collection

**Table:**

Oxford Diffraction Xcalibur Sapphire3 diffractometer	4744 independent reflections
Radiation source: fine-focus sealed tube	3408 reflections with *I* > 2σ(*I*)
Graphite monochromator	*R*_int_ = 0.048
Detector resolution: 16.1049 pixels mm^-1^	θ_max_ = 26.0°, θ_min_ = 3.7°
w scans	*h* = −12→12
Absorption correction: multi-scan (*CrysAlis PRO*; Oxford Diffraction, 2010)	*k* = −21→21
*T*_min_ = 0.438, *T*_max_ = 0.668	*l* = −17→17
26979 measured reflections	

### Refinement

**Table:**

Refinement on *F*^2^	Primary atom site location: structure-invariant direct methods
Least-squares matrix: full	Secondary atom site location: difference Fourier map
*R*[*F*^2^ > 2σ(*F*^2^)] = 0.045	Hydrogen site location: inferred from neighbouring sites
*wR*(*F*^2^) = 0.117	H-atom parameters constrained
*S* = 1.03	*w* = 1/[σ^2^(*F*_o_^2^) + (0.0505*P*)^2^ + 1.7709*P*] where *P* = (*F*_o_^2^ + 2*F*_c_^2^)/3
4744 reflections	(Δ/σ)_max_ = 0.001
299 parameters	Δρ_max_ = 0.42 e Å^−^^3^
0 restraints	Δρ_min_ = −0.65 e Å^−^^3^

### Special details

**Table:**

Experimental. Absorption correction: *CrysAlis PRO*, Oxford Diffraction Ltd., Version 1.171.34.40 (release 27–08-2010 CrysAlis171. NET) (compiled Aug 27 2010,11:50:40) Empirical absorption correction using spherical harmonics, implemented in SCALE3 ABSPACK scaling algorithm.
Geometry. All e.s.d.'s (except the e.s.d. in the dihedral angle between two l.s. planes) are estimated using the full covariance matrix. The cell e.s.d.'s are taken into account individually in the estimation of e.s.d.'s in distances, angles and torsion angles; correlations between e.s.d.'s in cell parameters are only used when they are defined by crystal symmetry. An approximate (isotropic) treatment of cell e.s.d.'s is used for estimating e.s.d.'s involving l.s. planes.
Refinement. Refinement of *F*^2^ against ALL reflections. The weighted *R*-factor *wR* and goodness of fit *S* are based on *F*^2^, conventional *R*-factors *R* are based on *F*, with *F* set to zero for negative *F*^2^. The threshold expression of *F*^2^ > σ(*F*^2^) is used only for calculating *R*-factors(gt) *etc*. and is not relevant to the choice of reflections for refinement. *R*-factors based on *F*^2^ are statistically about twice as large as those based on *F*, and *R*- factors based on ALL data will be even larger.

### Fractional atomic coordinates and isotropic or equivalent isotropic displacement parameters (Å^2^)

**Table:**

	*x*	*y*	*z*	*U*_iso_*/*U*_eq_	
O1	0.5036 (3)	0.16095 (19)	0.0690 (2)	0.0825 (9)	
O2	0.6714 (4)	0.24236 (18)	−0.00871 (19)	0.0816 (9)	
Br1	0.66487 (4)	0.12100 (3)	0.53546 (3)	0.06449 (16)	
S2	0.69475 (8)	0.25466 (5)	0.37229 (6)	0.0451 (2)	
S1	0.62576 (11)	0.20307 (6)	0.07413 (7)	0.0605 (3)	
Cl1	1.06308 (15)	−0.03829 (9)	0.18890 (14)	0.1136 (5)	
C1	0.7553 (4)	0.1383 (2)	0.1103 (3)	0.0516 (9)	
C2	0.8716 (5)	0.1366 (3)	0.0617 (3)	0.0750 (12)	
H2	0.8858	0.1717	0.0125	0.090*	
C3	0.9679 (5)	0.0824 (3)	0.0860 (4)	0.0851 (14)	
H3	1.0470	0.0803	0.0531	0.102*	
C4	0.9451 (4)	0.0318 (3)	0.1593 (4)	0.0711 (12)	
C5	0.8317 (4)	0.0349 (2)	0.2101 (3)	0.0647 (11)	
H5	0.8197	0.0012	0.2611	0.078*	
C6	0.7347 (4)	0.0882 (2)	0.1854 (3)	0.0582 (10)	
H6	0.6561	0.0903	0.2190	0.070*	
N7	0.6095 (3)	0.26564 (17)	0.1598 (2)	0.0492 (7)	
H7	0.5343	0.2686	0.1876	0.059*	
C8	0.7152 (3)	0.31610 (19)	0.1908 (2)	0.0405 (7)	
C9	0.7703 (4)	0.3677 (2)	0.1266 (3)	0.0503 (9)	
H9	0.7367	0.3699	0.0636	0.060*	
C10	0.8737 (4)	0.4154 (2)	0.1548 (3)	0.0509 (9)	
H10	0.9106	0.4487	0.1105	0.061*	
C11	0.9235 (3)	0.41437 (19)	0.2487 (3)	0.0461 (8)	
C12	0.8665 (3)	0.36557 (19)	0.3139 (2)	0.0431 (8)	
H12	0.8964	0.3659	0.3779	0.052*	
C13	0.7647 (3)	0.31593 (18)	0.2847 (2)	0.0382 (7)	
O14	1.0244 (3)	0.46473 (15)	0.2706 (2)	0.0633 (7)	
C15	1.0716 (5)	0.4676 (3)	0.3673 (4)	0.0833 (14)	
H15A	0.9983	0.4774	0.4083	0.125*	
H15B	1.1362	0.5083	0.3748	0.125*	
H15C	1.1123	0.4191	0.3845	0.125*	
C16	0.8284 (3)	0.18979 (17)	0.3927 (2)	0.0365 (7)	
N17	0.9356 (2)	0.19637 (14)	0.33991 (18)	0.0367 (6)	
C18	1.0363 (3)	0.14619 (18)	0.3584 (2)	0.0412 (8)	
N19	1.0348 (3)	0.08998 (17)	0.4253 (2)	0.0555 (8)	
C20	0.9257 (4)	0.0853 (2)	0.4755 (3)	0.0574 (10)	
H20	0.9215	0.0474	0.5227	0.069*	
C21	0.8188 (3)	0.13305 (19)	0.4616 (2)	0.0424 (8)	
N22	1.1446 (3)	0.15306 (17)	0.3042 (2)	0.0521 (7)	
C23	1.2648 (4)	0.1067 (3)	0.3206 (4)	0.0713 (12)	
H23A	1.2512	0.0708	0.3729	0.086*	
H23B	1.2824	0.0769	0.2634	0.086*	
C24	1.3790 (4)	0.1560 (3)	0.3443 (4)	0.0766 (13)	
H24A	1.3669	0.1796	0.4066	0.092*	
H24B	1.4591	0.1246	0.3486	0.092*	
O25	1.3958 (3)	0.2151 (2)	0.2743 (2)	0.0899 (11)	
C26	1.2790 (4)	0.2613 (3)	0.2621 (4)	0.0846 (15)	
H26A	1.2934	0.2995	0.2125	0.101*	
H26B	1.2632	0.2886	0.3215	0.101*	
C27	1.1609 (4)	0.2154 (3)	0.2354 (3)	0.0649 (11)	
H27A	1.0824	0.2482	0.2343	0.078*	
H27B	1.1704	0.1940	0.1716	0.078*	

### Atomic displacement parameters (Å^2^)

**Table:**

	*U*^11^	*U*^22^	*U*^33^	*U*^12^	*U*^13^	*U*^23^
O1	0.0727 (19)	0.083 (2)	0.089 (2)	−0.0082 (16)	−0.0313 (16)	−0.0141 (18)
O2	0.127 (3)	0.079 (2)	0.0388 (15)	0.0099 (19)	−0.0027 (15)	0.0047 (14)
Br1	0.0599 (3)	0.0763 (3)	0.0583 (3)	−0.0100 (2)	0.01681 (19)	0.0202 (2)
S2	0.0322 (4)	0.0552 (5)	0.0482 (5)	0.0007 (4)	0.0061 (3)	0.0165 (4)
S1	0.0729 (7)	0.0615 (6)	0.0461 (5)	−0.0010 (5)	−0.0126 (5)	−0.0021 (5)
Cl1	0.0878 (9)	0.0894 (10)	0.1623 (15)	0.0286 (8)	−0.0144 (9)	−0.0113 (9)
C1	0.058 (2)	0.049 (2)	0.048 (2)	−0.0077 (17)	−0.0057 (17)	−0.0081 (17)
C2	0.082 (3)	0.076 (3)	0.068 (3)	−0.001 (3)	0.018 (2)	0.006 (2)
C3	0.069 (3)	0.091 (4)	0.096 (4)	0.000 (3)	0.018 (3)	−0.014 (3)
C4	0.064 (3)	0.058 (3)	0.091 (3)	0.003 (2)	−0.006 (2)	−0.014 (2)
C5	0.064 (3)	0.048 (2)	0.082 (3)	−0.010 (2)	−0.007 (2)	0.005 (2)
C6	0.057 (2)	0.054 (2)	0.063 (2)	−0.0094 (19)	−0.0005 (19)	0.001 (2)
N7	0.0450 (16)	0.0564 (18)	0.0462 (16)	−0.0007 (14)	−0.0001 (13)	0.0004 (14)
C8	0.0393 (17)	0.0421 (19)	0.0405 (18)	0.0066 (14)	0.0061 (14)	0.0035 (14)
C9	0.063 (2)	0.052 (2)	0.0365 (18)	0.0063 (18)	0.0070 (16)	0.0057 (16)
C10	0.057 (2)	0.045 (2)	0.052 (2)	0.0012 (17)	0.0192 (17)	0.0131 (17)
C11	0.0397 (18)	0.0387 (19)	0.060 (2)	0.0016 (15)	0.0103 (16)	0.0091 (16)
C12	0.0398 (17)	0.044 (2)	0.0454 (19)	0.0028 (15)	−0.0019 (15)	0.0102 (15)
C13	0.0346 (16)	0.0376 (17)	0.0426 (18)	0.0048 (13)	0.0061 (14)	0.0103 (14)
O14	0.0573 (15)	0.0597 (16)	0.0730 (19)	−0.0169 (13)	0.0053 (14)	0.0132 (14)
C15	0.073 (3)	0.086 (3)	0.089 (3)	−0.032 (3)	−0.016 (3)	0.017 (3)
C16	0.0339 (16)	0.0369 (17)	0.0384 (17)	−0.0049 (13)	−0.0027 (13)	0.0033 (14)
N17	0.0300 (13)	0.0380 (15)	0.0420 (15)	−0.0040 (11)	−0.0004 (11)	0.0057 (12)
C18	0.0352 (17)	0.0381 (18)	0.050 (2)	−0.0063 (14)	−0.0007 (15)	0.0013 (15)
N19	0.0500 (17)	0.0424 (17)	0.074 (2)	0.0040 (14)	0.0052 (16)	0.0190 (15)
C20	0.060 (2)	0.047 (2)	0.065 (2)	−0.0005 (18)	0.0028 (19)	0.0229 (19)
C21	0.0440 (18)	0.0411 (19)	0.0425 (19)	−0.0075 (15)	0.0064 (15)	0.0137 (15)
N22	0.0364 (15)	0.0505 (18)	0.070 (2)	0.0009 (13)	0.0085 (14)	0.0098 (16)
C23	0.048 (2)	0.067 (3)	0.099 (3)	0.015 (2)	0.013 (2)	0.000 (2)
C24	0.042 (2)	0.092 (3)	0.095 (3)	−0.005 (2)	−0.006 (2)	0.032 (3)
O25	0.0371 (14)	0.136 (3)	0.096 (2)	−0.0163 (16)	−0.0023 (14)	0.056 (2)
C26	0.064 (3)	0.101 (4)	0.088 (3)	−0.025 (3)	−0.010 (2)	0.043 (3)
C27	0.040 (2)	0.095 (3)	0.060 (2)	−0.003 (2)	0.0070 (17)	0.017 (2)

### Geometric parameters (Å, º)

**Table:**

O1—S1	1.426 (3)	C12—C13	1.386 (4)
O2—S1	1.427 (3)	C12—H12	0.9300
Br1—C21	1.894 (3)	O14—C15	1.414 (5)
S2—C16	1.765 (3)	C15—H15A	0.9600
S2—C13	1.777 (3)	C15—H15B	0.9600
S1—N7	1.623 (3)	C15—H15C	0.9600
S1—C1	1.777 (4)	C16—N17	1.326 (4)
Cl1—C4	1.736 (5)	C16—C21	1.379 (4)
C1—C2	1.367 (6)	N17—C18	1.351 (4)
C1—C6	1.379 (5)	C18—N19	1.347 (4)
C2—C3	1.381 (7)	C18—N22	1.348 (4)
C2—H2	0.9300	N19—C20	1.320 (5)
C3—C4	1.368 (7)	C20—C21	1.363 (5)
C3—H3	0.9300	C20—H20	0.9300
C4—C5	1.360 (6)	N22—C27	1.455 (5)
C5—C6	1.376 (6)	N22—C23	1.461 (5)
C5—H5	0.9300	C23—C24	1.458 (6)
C6—H6	0.9300	C23—H23A	0.9700
N7—C8	1.430 (4)	C23—H23B	0.9700
N7—H7	0.8600	C24—O25	1.426 (5)
C8—C13	1.384 (4)	C24—H24A	0.9700
C8—C9	1.391 (5)	C24—H24B	0.9700
C9—C10	1.374 (5)	O25—C26	1.424 (5)
C9—H9	0.9300	C26—C27	1.465 (6)
C10—C11	1.384 (5)	C26—H26A	0.9700
C10—H10	0.9300	C26—H26B	0.9700
C11—O14	1.363 (4)	C27—H27A	0.9700
C11—C12	1.378 (4)	C27—H27B	0.9700
			
C16—S2—C13	100.11 (14)	O14—C15—H15B	109.5
O1—S1—O2	120.05 (19)	H15A—C15—H15B	109.5
O1—S1—N7	105.76 (18)	O14—C15—H15C	109.5
O2—S1—N7	108.56 (17)	H15A—C15—H15C	109.5
O1—S1—C1	108.13 (18)	H15B—C15—H15C	109.5
O2—S1—C1	106.19 (19)	N17—C16—C21	121.5 (3)
N7—S1—C1	107.62 (16)	N17—C16—S2	118.8 (2)
C2—C1—C6	120.8 (4)	C21—C16—S2	119.7 (2)
C2—C1—S1	120.1 (3)	C16—N17—C18	117.0 (3)
C6—C1—S1	119.1 (3)	N19—C18—N22	118.5 (3)
C1—C2—C3	119.6 (4)	N19—C18—N17	124.9 (3)
C1—C2—H2	120.2	N22—C18—N17	116.6 (3)
C3—C2—H2	120.2	C20—N19—C18	116.0 (3)
C4—C3—C2	119.0 (4)	N19—C20—C21	123.3 (3)
C4—C3—H3	120.5	N19—C20—H20	118.3
C2—C3—H3	120.5	C21—C20—H20	118.3
C5—C4—C3	121.7 (4)	C20—C21—C16	117.3 (3)
C5—C4—Cl1	118.6 (4)	C20—C21—Br1	120.5 (2)
C3—C4—Cl1	119.7 (4)	C16—C21—Br1	122.2 (2)
C4—C5—C6	119.6 (4)	C18—N22—C27	122.9 (3)
C4—C5—H5	120.2	C18—N22—C23	122.7 (3)
C6—C5—H5	120.2	C27—N22—C23	113.7 (3)
C5—C6—C1	119.3 (4)	C24—C23—N22	110.6 (4)
C5—C6—H6	120.4	C24—C23—H23A	109.5
C1—C6—H6	120.4	N22—C23—H23A	109.5
C8—N7—S1	122.6 (2)	C24—C23—H23B	109.5
C8—N7—H7	118.7	N22—C23—H23B	109.5
S1—N7—H7	118.7	H23A—C23—H23B	108.1
C13—C8—C9	118.1 (3)	O25—C24—C23	112.0 (4)
C13—C8—N7	121.6 (3)	O25—C24—H24A	109.2
C9—C8—N7	120.3 (3)	C23—C24—H24A	109.2
C10—C9—C8	120.9 (3)	O25—C24—H24B	109.2
C10—C9—H9	119.5	C23—C24—H24B	109.2
C8—C9—H9	119.5	H24A—C24—H24B	107.9
C9—C10—C11	120.6 (3)	C26—O25—C24	111.7 (3)
C9—C10—H10	119.7	O25—C26—C27	112.4 (4)
C11—C10—H10	119.7	O25—C26—H26A	109.1
O14—C11—C12	124.5 (3)	C27—C26—H26A	109.1
O14—C11—C10	116.4 (3)	O25—C26—H26B	109.1
C12—C11—C10	119.0 (3)	C27—C26—H26B	109.1
C11—C12—C13	120.3 (3)	H26A—C26—H26B	107.8
C11—C12—H12	119.9	N22—C27—C26	110.0 (3)
C13—C12—H12	119.9	N22—C27—H27A	109.7
C8—C13—C12	121.0 (3)	C26—C27—H27A	109.7
C8—C13—S2	120.8 (2)	N22—C27—H27B	109.7
C12—C13—S2	118.1 (2)	C26—C27—H27B	109.7
C11—O14—C15	117.3 (3)	H27A—C27—H27B	108.2
O14—C15—H15A	109.5		
			
O1—S1—C1—C2	132.3 (3)	C11—C12—C13—S2	−179.8 (2)
O2—S1—C1—C2	2.2 (4)	C16—S2—C13—C8	111.6 (3)
N7—S1—C1—C2	−113.9 (3)	C16—S2—C13—C12	−70.9 (3)
O1—S1—C1—C6	−45.3 (3)	C12—C11—O14—C15	−1.2 (5)
O2—S1—C1—C6	−175.4 (3)	C10—C11—O14—C15	176.6 (4)
N7—S1—C1—C6	68.5 (3)	C13—S2—C16—N17	−4.3 (3)
C6—C1—C2—C3	2.1 (6)	C13—S2—C16—C21	176.6 (3)
S1—C1—C2—C3	−175.5 (4)	C21—C16—N17—C18	−1.6 (4)
C1—C2—C3—C4	−0.6 (7)	S2—C16—N17—C18	179.3 (2)
C2—C3—C4—C5	−1.6 (7)	C16—N17—C18—N19	0.8 (5)
C2—C3—C4—Cl1	178.4 (4)	C16—N17—C18—N22	179.7 (3)
C3—C4—C5—C6	2.4 (7)	N22—C18—N19—C20	−179.2 (3)
Cl1—C4—C5—C6	−177.6 (3)	N17—C18—N19—C20	−0.3 (5)
C4—C5—C6—C1	−0.9 (6)	C18—N19—C20—C21	0.6 (6)
C2—C1—C6—C5	−1.3 (6)	N19—C20—C21—C16	−1.4 (6)
S1—C1—C6—C5	176.3 (3)	N19—C20—C21—Br1	178.9 (3)
O1—S1—N7—C8	176.2 (3)	N17—C16—C21—C20	1.9 (5)
O2—S1—N7—C8	−53.7 (3)	S2—C16—C21—C20	−179.0 (3)
C1—S1—N7—C8	60.8 (3)	N17—C16—C21—Br1	−178.3 (2)
S1—N7—C8—C13	−121.0 (3)	S2—C16—C21—Br1	0.8 (4)
S1—N7—C8—C9	59.7 (4)	N19—C18—N22—C27	−175.0 (3)
C13—C8—C9—C10	2.0 (5)	N17—C18—N22—C27	6.0 (5)
N7—C8—C9—C10	−178.7 (3)	N19—C18—N22—C23	−5.6 (5)
C8—C9—C10—C11	−1.4 (5)	N17—C18—N22—C23	175.4 (3)
C9—C10—C11—O14	−178.8 (3)	C18—N22—C23—C24	−118.5 (4)
C9—C10—C11—C12	−1.0 (5)	C27—N22—C23—C24	51.7 (5)
O14—C11—C12—C13	−179.6 (3)	N22—C23—C24—O25	−53.3 (5)
C10—C11—C12—C13	2.8 (5)	C23—C24—O25—C26	56.6 (6)
C9—C8—C13—C12	−0.2 (5)	C24—O25—C26—C27	−56.8 (5)
N7—C8—C13—C12	−179.5 (3)	C18—N22—C27—C26	118.8 (4)
C9—C8—C13—S2	177.3 (2)	C23—N22—C27—C26	−51.5 (5)
N7—C8—C13—S2	−2.0 (4)	O25—C26—C27—N22	53.5 (5)
C11—C12—C13—C8	−2.2 (5)		

### Hydrogen-bond geometry (Å, º)

**Table:**

*D*—H···*A*	*D*—H	H···*A*	*D*···*A*	*D*—H···*A*
N7—H7···O25^i^	0.86	2.09	2.849 (4)	148
C20—H20···N19^ii^	0.93	2.52	3.352 (5)	149

Symmetry codes: (i) *x*−1, *y*, *z*; (ii) −*x*+2, −*y*, −*z*+1.
